# Optimal view detection for ultrasound-guided supraclavicular block using deep learning approaches

**DOI:** 10.1038/s41598-023-44170-y

**Published:** 2023-10-11

**Authors:** Yumin Jo, Dongheon Lee, Donghyeon Baek, Bo Kyung Choi, Nisan Aryal, Jinsik Jung, Yong Sup Shin, Boohwi Hong

**Affiliations:** 1 Department of Anaesthesiology and Pain Medicine, College of Medicine, Chungnam National University and Hospital, 282 Munhwar-ro, Jung-gu, Daejeon, 35015 Republic of Korea; 2 Department of Biomedical Engineering, College of Medicine, Chungnam National University and Hospital, Daejeon, Republic of Korea; 3 Chungnam National University College of Medicine, Daejeon, Republic of Korea; 4 MTEG Co., Ltd, Seoul, Republic of Korea; 5 Biomedical Research Institute, Chungnam National University Hospital, Daejeon, Republic of Korea

**Keywords:** Biomedical engineering, Preclinical research

## Abstract

Successful ultrasound-guided supraclavicular block (SCB) requires the understanding of sonoanatomy and identification of the optimal view. Segmentation using a convolutional neural network (CNN) is limited in clearly determining the optimal view. The present study describes the development of a computer-aided diagnosis (CADx) system using a CNN that can determine the optimal view for complete SCB in real time. The aim of this study was the development of computer-aided diagnosis system that aid non-expert to determine the optimal view for complete supraclavicular block in real time. Ultrasound videos were retrospectively collected from 881 patients to develop the CADx system (600 to the training and validation set and 281 to the test set). The CADx system included classification and segmentation approaches, with Residual neural network (ResNet) and U-Net, respectively, applied as backbone networks. In the classification approach, an ablation study was performed to determine the optimal architecture and improve the performance of the model. In the segmentation approach, a cascade structure, in which U-Net is connected to ResNet, was implemented. The performance of the two approaches was evaluated based on a confusion matrix. Using the classification approach, ResNet34 and gated recurrent units with augmentation showed the highest performance, with average accuracy 0.901, precision 0.613, recall 0.757, f1-score 0.677 and AUROC 0.936. Using the segmentation approach, U-Net combined with ResNet34 and augmentation showed poorer performance than the classification approach. The CADx system described in this study showed high performance in determining the optimal view for SCB. This system could be expanded to include many anatomical regions and may have potential to aid clinicians in real-time settings.

*Trial registration* The protocol was registered with the Clinical Trial Registry of Korea (KCT0005822, https://cris.nih.go.kr).

## Introduction

Supraclavicular block (SCB) is useful for both surgical anesthesia and perioperative analgesia in patients undergoing upper limb surger^1^. Ultrasound-guided SCB was shown to be safer than landmark or nerve stimulator techniques, as it alleviates concerns about the proximity of the brachial plexus (BP) to the pleura and reduces inadvertent vascular punctures^2,3^. Ultrasound can easily visualize the BP, lying close (postero-lateral) to the pulsatile subclavian artery (SA) above the hyperechoic first rib at the supraclavicular fossa^4^. In this position, all the components of the BP are surrounded by a sheath at a shallow depth just above the clavicle. SCB, also called the ‘spinal of the arm’, can therefore anesthetize almost the entire upper extremity^1^.

However, simply using ultrasound alone does not guarantee the success of SCB^2^ or avoid complications such as pneumothorax^5^. Optimising the ultrasound view for SCB requires the probe should be angled or tilted until a lower trunk is visualized laterally to the SA and above the clavicle^6^. This so-called corner pocket (CP) approach^7^ is a common and safe procedure with a low risk of having pneumothorax^8^; however, few studies have focused on classifying the optimal view for SCB.

Ultrasound-guided regional anesthesia (UGRA) has been a useful technique for perioperative anesthesia as it can prevent inadvertent injuries caused by needles^1^. Recently, AI-assisted UGRA has become a novel technology that aids the process of local anesthetic injection to peripheral nerves. The greatest advantage of a deep learning-based computer-aided diagnosis (CADx) system is that it is suitable for the increasing demand for training UGRA for residents^9–11^. Feedback by the CADx system can be helpful for learners who do not have much access to guided training^12,13^. Bowness, James et al. developed a U-Net based ScanNav that performs segmentation on seven different anatomical structures, including the supraclavicular region, using 253 scan videos obtained from 244 healthy individuals^14^. Notably, ScanNav visualizes a color overlay on real-time ultrasound to highlight key anatomical structures, specifically, brachial plexus (BP) region. It may support non-experts in training and clinical practice, and experts in teaching UGRA^15^. However, the issue of clinicians having to perform time-consuming labelling work remains a challenge for conducting the segmentation task.

Tyagi et al. developed a segmentation model with dice coefficient performances of 0.84 and 0.94 in the Supraclavicular and interscalene areas, respectively, using approximately 35,000 frames of ultrasound images obtained from 196 patients^16^. However, while showing high performance on images from a single ultrasound device, the model has limitations in its generality, as it performs poorly on lower quality images from different ultrasound devices.

To handle current limitations on AI-assisted UGRA for SCB, we suggest a deep learning-based CADx system that can distinguish between optimal and non-optimal views for the CP approach. The CADx systems were developed with a classification approach that trains all ultrasound images, distinguishes between optimal and non-optimal images, and a segmentation approach that trains images by designating anatomical structures, as in previous studies. Our CADx system showed considerable accuracy and robustness across three ultrasound machines. Furthermore, it can visualize the optimality score and semantic segmentation results in a real-time environment.

## Methods

### Study design

**Figure 1:**
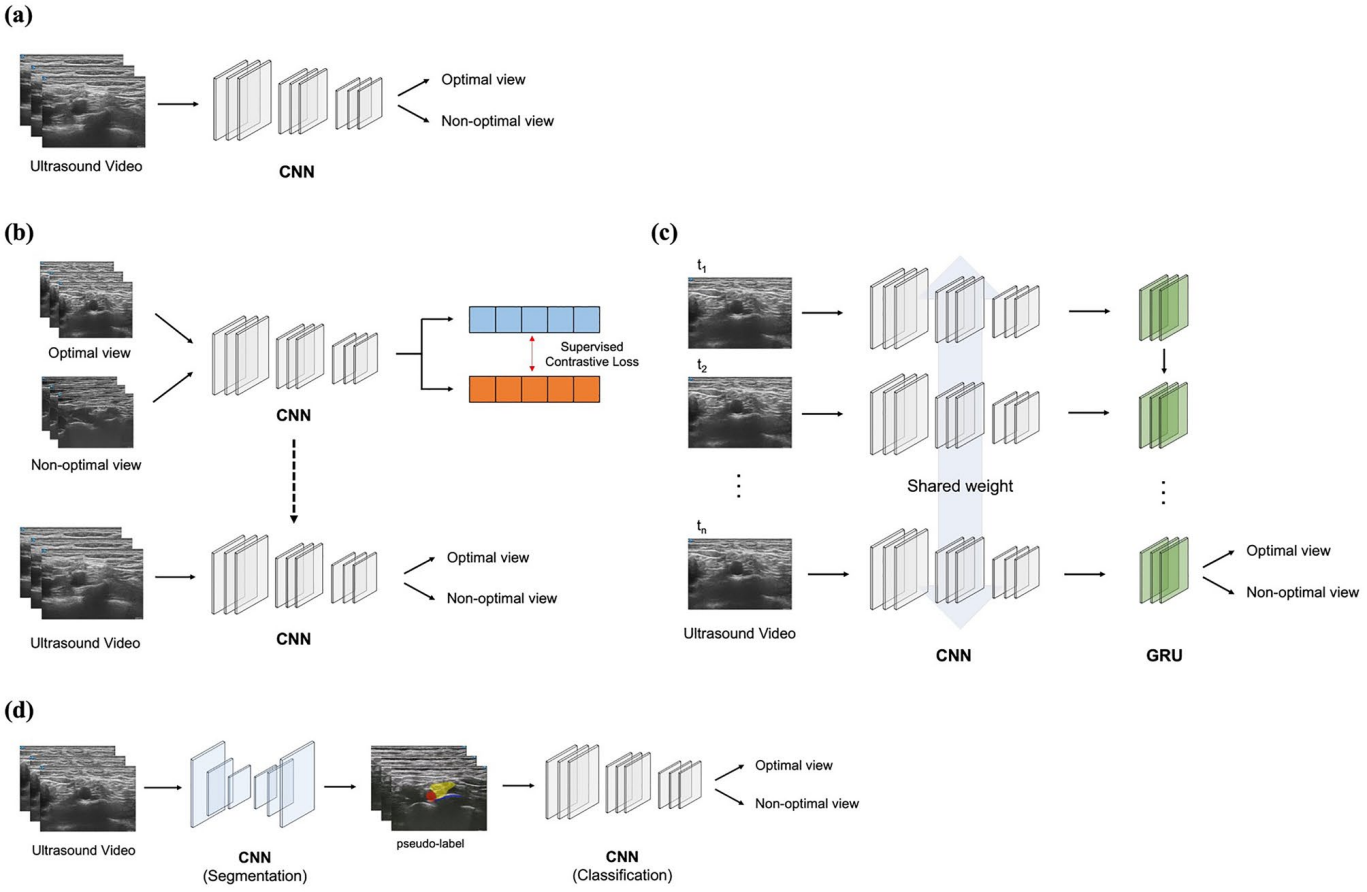
Overview of computer-aided diagnosis systems for determining the optimal view for ultrasound-guided supraclavicular block. (**a**) Classification approach: Vanilla CNN (**b**) classification approach: supervised contrastive learning (**c**) classification approach: CNN combined with GRU (**d**) segmentation approach; the predicted segmentation maps from the segmentation model act as pseudo-labels and serve as inputs for the subsequent classification model.

The CADx systems were developed using a convolutional neural network (CNN) to detect the optimal view for ultrasound-guided SCB. The developed systems utilized a classification approach and a segmentation approach (Fig. 1). This single-centre, retrospective study was approved by the institutional review board of Chungnam National University Hospital (CNUH, Daejeon, Korea, CNUH IRB 2022-05-071, Chairperson Prof. Jeong Lan Kim) on 10 June 2022, with the modified protocol approved on 7 November 2022. Due to the retrospective nature of the study, CNUH waived the need of obtaining informed consent. The protocol was registered with the Clinical Trial Registry of Korea (KCT0007482, https://cris.nih.go.kr). All experiments were performed in accordance with relevant guidelines and regulations.

### Ultrasound image acquisition and curation

All patients at CNUH underwent routine sequential ultrasound imaging technique (SUIT) before any BP block^6^, as SUIT has been shown useful to identify individual elements of the BP and vascular structures above the clavicle^17^. Patients were maintained in a supine position with the head turned to the contralateral side and the ipsilateral shoulder slightly elevated with a pillow (Supplementary Fig. 1a). The probe was applied to the upper part of the interscalene groove and slid downward from the fifth cervical nerve root (C5) to the first thoracic nerve root (T1) until the complete BP was evaluated on the first rib at the supraclavicular fossa; the probe was subsequently slid upward in the reverse direction to the interscalene level^6^.

The optimal image for each SCB was defined as an image that enabled visualization of the corner pocket by the first rib, the SA and the neural component (Supplementary Fig. 1b)^7^. Optimal and non-optimal images were distinguished by several ultrasound anatomic characteristics. In optimal images, the SA presented large and round hypoechoic appearance with clear hyperechoic rim. In addition, while sliding the probe from the cephalad to caudad direction, the first rib was visible from lateral to medial under the BP. The BP appeared as a honeycomb mass or small hypoechoic clustered structure and was located on the inferolateral side of the SA and completely above the first rib. Both the nerve itself and the relationship between each BP and its adjacent structures were important in determining the optimal view^6^.

Patient data at CNUH were collected using a video capture device (SurgBox, MTEG Co. Ltd. Korea) and stored on a video archiving and communication system (VACS; MTEG Co. Ltd.)^18^. The ultrasound videos were reviewed by two regional anaesthesiologists, who distinguished between optimal and non-optimal imaging datasets and selected well-focused, high-quality videos of appropriate brightness and depth. All patient-identifying information on ultrasound images was pseudonymized.

### Dataset

Ultrasound videos were collected retrospectively from 881 patients scheduled to undergo elective surgery at CNUH from January 2019 to August 2022. Videos of image quality too poor to detect BP were excluded. The images were sampled from the videos to construct datasets for training, validation, and test steps. The sampling rate was a half second which is lower than the minimal unit of the labeled classification interval.

The method proposed in this study consists of classification and segmentation approaches. For the training and validation datasets, the former used all frames as labels, while the latter used masks from a single frame of the optimal view as labels. Therefore, in the classification approach, the labels consist of frames from both optimal and non-optimal views in the videos, and in the segmentation approach, masks from one section of the optimal view are used as labels, which corresponds to the number of ultrasound videos.

The training and validation dataset consisted of 600 ultrasound videos obtained with a high-resolution ultrasound system (X-Porte, FUJIFILM SonoSite, Inc., Bothell, USA) and HFL50xp 15–6 MHz probe (X-Porte). The training set consisted of 3060 optimal images and 28,085 non-optimal images from 506 videos. The validation set consisted of 618 optimal images and 5799 non-optimal images from 94 videos (Supplementary Fig. 2).

Three test datasets were prepared. For generalizability, two datasets were constructed from images taken by ultrasound machines different from the training dataset. The test set 1 consisted of 1030 optimal images and 4532 non-optimal images from 100 videos obtained from an additional 100 patients using the X-Porte ultrasound system. The test set 2 consisted of 836 optimal images and 7718 non-optimal images from 100 videos obtained from 100 patients with a Venue Go ultrasound machine (GE Healthcare, Florida, USA) and a 12–4 MHz linear probe. The test set 3 consisted of 755 optimal images and 5032 non-optimal images obtained from 81 videos of 81 patients with a TE 7 ultrasound machine (Mindray, Shenzhen, China) and a 11–3 MHz linear probe. The compositions of these datasets, including the demographic characteristics of included patients, are shown in Table 1.

**Table 1:** Dataset composition and demographics.

Dataset	Training (Cls./Seg.)	Validation (Cls./Seg.)	Test 1 (X-Porte)	Test 2 (Venue Go)	Test 3 (TE7)
Number of Patients (videos)	506	94	100	100	81
Number of Images (frames)	31,145/506	6417/94	5562	8554	5787
Demographics					
Age (years)	55.0 [36.5;64.0]	57.5 [43.0;67.0]	57.0 [42.5;65.0]	62.0 [53.5;70.5]	64.0 [54.0;74.0]
Female (n)	225 (44.8%)	41 (43.6%)	44 (44.4%)	48 (48.5%)	43 (54.4%)
Height (cm)	164.0 [156.0;171.0]	162.5 [156.0;170.0]	162.0 [155.0;170.0]	161.0 [156.0;168.0]	159.0 [153.0;170.0]
Body weight (kg)	64.5 [56.0;74.0]	64.0 [58.0;72.0]	66.0 [58.5;73.5]	60.0 [53.5;68.5]	62.5 [56.0;71.5]
BMI (kg/m^2^)	24.2 [22.1;26.7]	24.4 [22.9;26.8]	24.8 [22.3;27.0]	23.1 [20.6;26.0]	24.5 [22.5;27.2]

Values are presented as number (%) or median [interquartile range].

*Cls* classification, *Seg* segmentation.

### Preprocessing

The images from the datasets were processed before being fed into the CADx system. For the CNN training, the acquired ultrasound images were resized to 224 × 224 pixels. Adapted from the work by Pi et al.^19^, we designed an automated background removal algorithm that can be deployed in a real-time setting (Supplementary Fig. 6). To improve performance during the training phase, augmentation techniques, such as random cropping, noise addition, blurring, affine transformation, non-linear spatial transformation, and adjusting brightness and contrast were randomly applied. The software used for image preprocessing was OpenCV (version 4.5.5.64) and an image augmentation library called Albumentations^20^. Examples of the augmentation results are shown in Supplementary Fig. 3.

### Development of CNN for determining optimal views: classification approach

Residual neural network (ResNet) was selected for the backbone network of the CADx system for its excellence in classification tasks despite of its relatively small model size. The performances of the ResNet models were compared by training various models from shallow to deep layers.

The ResNet model has a CNN encoder part and a fully connected (FC) layer. An optimality score for SCB is then computed from the output of the FC layer normalized by a sigmoid function^21^. As proposed by Van Boxtel et al.^22^, ResNet (18, 34, 50, 101, and 152 layers) models, pre-trained with the ImageNet dataset, were trained with a binary cross entropy loss. As the number of optimal view images was far less than the non-optimal view, the weight for the optimal view class was multiplied by the ratio of the number of optimal to non-optimal view examples. Among the ResNet models, the one that performed best according to our evaluation method was selected for further modifications to improve its classification performance.

#### Recurrent neural network

In another model, it was devised that the encoder branch of the ResNet was connected to a recurrent neural network (RNN) as shown in Fig. 1c. The features of sequential images extracted by the encoder were taken as serial inputs to RNN to capture time-dependent features^23^. For the classification task, only the output of the last RNN unit was used to classify its label (many-to-one). Similar to the proposed structure by Chen et al.^24^ where CNN was jointly combined with a long short-term memory (LSTM) network^25^, we adopted the gated recurrent units which are known to require less memory than LSTM but can deal with the vanishing gradient problem of a vanilla RNNs^26^. The extracted features were embedded into a latent space of 128 dimensions. The hidden dimension and the number of nodes of RNN were set to 256 and 8. Finally, an FC layer was joined to the output of the last node for the optimal view classification.

#### Supervised contrastive learning

One of our proposed networks utilized supervised contrastive learning^27^ before getting trained for the optimal view classification task as shown in Fig. 1b. We utilized the labels of data to better segregate the ultrasound images in a feature space similar in essence to self-supervised learning that learns the representations of unlabeled data^28^. Using the method in Khosla et al.^27^, the encoder network was pre-trained with the supervised contrastive learning loss, and then the linear classifier was jointly trained with the encoder.

### Development of CNN for determining optimal views: segmentation approach

The segmentation approach was a CADx system that predicts the regions of the SA, first rib and BP to determine whether the view is optimal or not. Because the optimal view could not be directly determined from the predicted result of the segmentation model, the classification model was applied as a cascaded structure after application of the semantic segmentation model. U-Net^29^, as a semantic segmentation network was combined with the ResNet encoder (Fig. 1d). This approach is similar to Van Boxtel et al.^22^, but basically different in that the segmentation network is followed by the classification network. The predicted segmentation maps, along with the original ultrasound images, were reused as input to the classification model, as pseudo-labels.

### Implementation details

All the models except for the segmentation network were trained maximum of 30 epochs, with an initial learning rate of 0.0001 with a decay of 0.1 for every 10 epochs. The segmentation network was trained maximum of 100 epochs, with an initial learning rate of 0.001 with a decay of 0.1 for every 25 epochs. In all cases, a batch size of 64 and an Adam optimizer were used^30^. The models were developed on an NVIDIA V100 GPU and were implanted by Python (version 3.9.; Python Software Foundation, Beaverton, OR) and PyTorch (version 1.11.0) software. All source codes of this study are uploaded on https://github.com/nistring/Ultrasound-Optimal-View-Detection.

### Evaluation metrics

On test datasets, the labeled ground truth was compared with the model’s prediction. Receiver operating characteristic (ROC) curves and precision-recall (PR) curves were plotted with the areas under the ROC (AUROC) and PR curves (AUPRC). Since AUPRC is more appropriate than AUROC when there’s a highly imbalanced dataset^31^, AUPRC was mainly used as a metric for comparing the models’ performances. Subsequently, accuracy, precision, recall, and f1-score were calculated on an optimality threshold value where the f1-score had the highest value on ROC curves. The Inference speed was measured by a unit of frames per second (fps).

**Figure 2:**
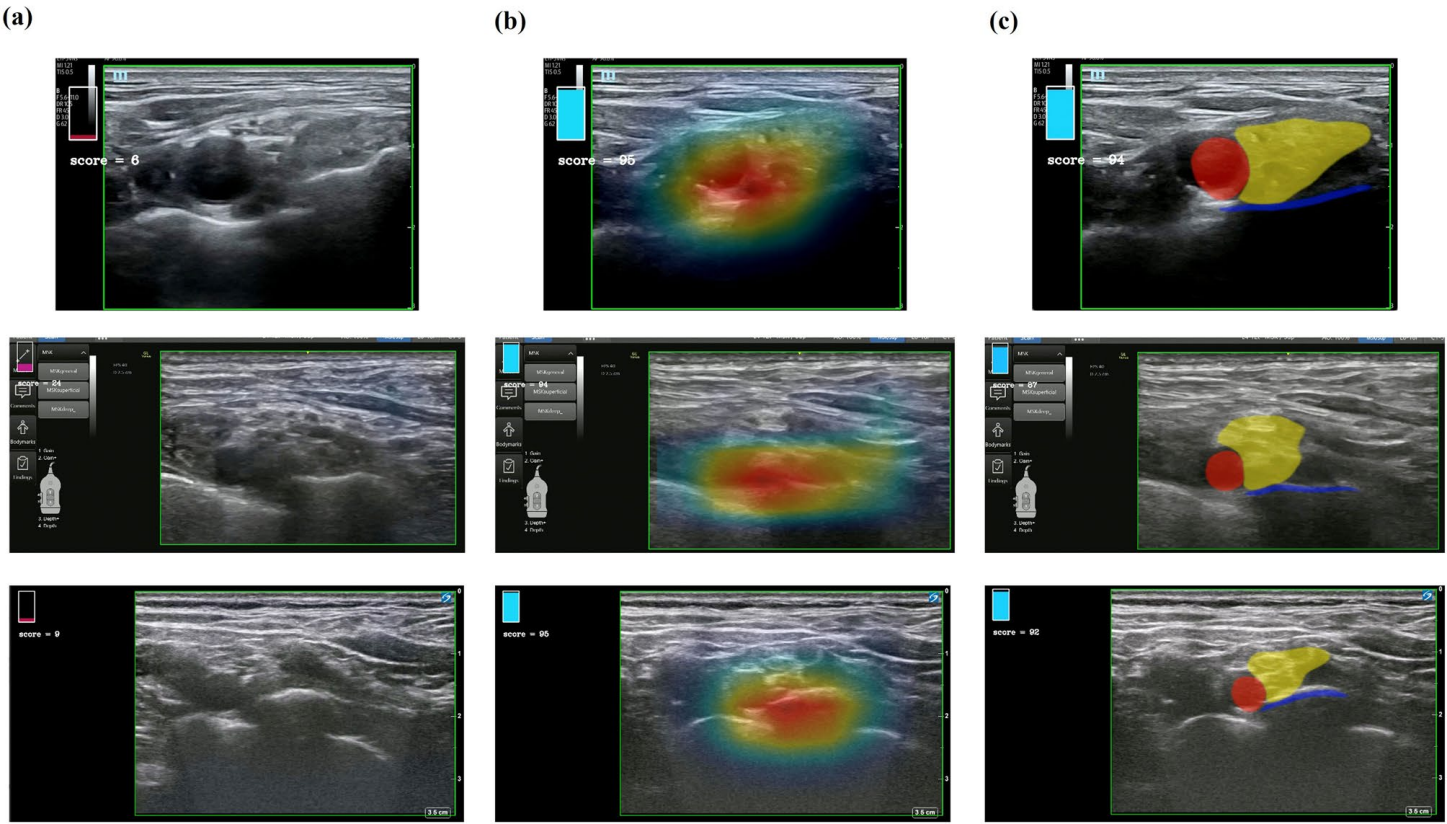
Qualitative results of deep learning approaches for determining optimal views for ultrasound-guided supraclavicular block. The bar at the top-left represents the probability predicted by the convolutional neural network model. TE7, Venue Go, and X-Porte results are pictured in order from top to bottom. (**a**) Original ultrasound images. (**b**) Results predicted by the classification approach: gradient-weighted class activation mapping. (**c**) Results predicted by the segmentation approach.

In the inference phase, gradient-weighted class activation mapping (Grad-CAM)^32^ was applied as described in Hassanien et al.^33^. The Grad-CAM was calculated by the weights of the last convolutional layer of CNN to identify the region within each ultrasound image that influenced the CNN's prediction (Fig. 2b).

## Results

### Comparative performances among classification approaches

In this work, we sought to discover the best backbone layer for predicting the optimal view for SCB among the ResNet with different number of layers. Comparing performances of ResNet with different number of layers (18, 34, 50, 101, and 152 layers), AUROC and AUPRC didn't significantly improve and began to level off for the networks deeper than ResNet34 (Supplementary Fig. 4).

**Figure 3:**
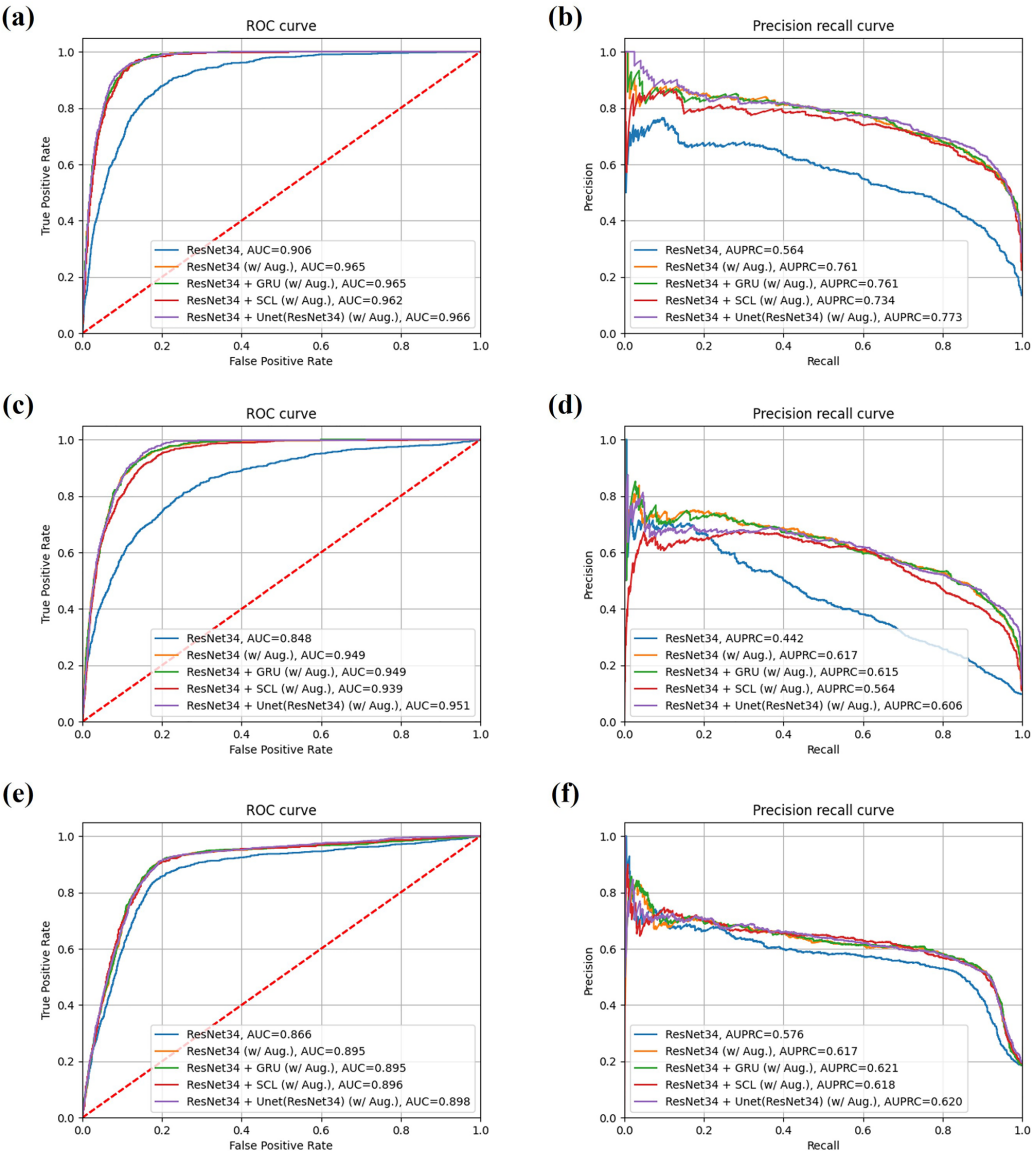
Comparative performances of the proposed deep learning approaches: (**a**, **c**, **e**) ROC curves of test sets (**a**) 1 (X-Porte), (**c**) 2 (Venue Go), and (**e**) 3 (TE7). (**b**, **d**, **f**) PR curves of test sets (**b**) 1 (X-Porte), (**d**) 2 (Venue Go), and (**f**) 3 (TE7).

Thus, ResNet34 was selected as the backbone model and several approaches was evaluated. (Table 2). The predicted probability on test-set lower than the threshold value was classified as non-optimal view. The threshold value was 0.81 obtained when the f1-score on validation set was highest. It is evident that the image augmentation made the model more generalizable to images from other US machines (Table 2; Fig. 3). ResNet34 with GRU and augmentation showed the highest performance, with a mean accuracy of 0.901, a mean precision of 0.613, a mean recall of 0.757, a mean f1-score of 0.677, and a mean AUROC of 0.936. This method, however, showed the lowest performance at 153.2 fps (Table 2; Fig. 3b).

**Table 2:** Comparative performances of deep learning approaches.

Deep learning approach	CNN model	Test set	Acc	Pre	Rec	F1-score	AUROC	AUPRC	fps
Classification	ResNet34	Test set 1 (X-Porte)	0.839	0.551	0.717	0.623	0.866	0.576	–
		Test set 2 (Venue Go)	0.859	0.37	0.627	0.466	0.848	0.442	–
		Test set 3 (TE7)	0.873	0.509	0.683	0.584	0.906	0.564	–
		Average	0.857	0.477	0.676	0.558	0.873	0.527	888.4
	ResNet34 (w/Aug.)	Test set 1 (X-Porte)	0.861	0.605	0.714	0.655	0.895	0.617	–
		Test set 2 (Venue Go)	0.91	0.57	0.688	0.623	0.949	0.617	–
		Test set 3 (TE7)	0.924	0.683	0.784	0.73	0.965	0.761	–
		Average	0.901	0.619	0.728	0.669	0.936	0.665	790.1
	ResNet34 + GRU (w/Aug.)	Test set 1 (X-Porte)	0.863	0.607	0.73	0.66	0.895	0.621	–
		Test set 2 (Venue Go)	0.917	0.558	0.724	0.63	0.949	0.615	–
		Test set 3 (TE7)	0.924	0.673	0.815	0.737	0.965	0.761	–
		Average	0.901	0.613	**0.757**	**0.677**	0.936	**0.666**	153.2
	ResNet34 + SCL (w/Aug.)	Test set 1 (X-Porte)	0.858	0.596	0.726	0.655	0.896	0.618	–
		Test set 2 (Venue Go)	0.915	0.551	0.69	0.614	0.939	0.564	–
		Test set 3 (TE7)	0.923	0.67	0.8	0.729	0.962	0.734	–
		Average	0.899	0.606	0.74	0.666	0.932	0.639	**910.8**
Segmentation	U-Net + ResNet34 (w/Aug.)	Test set 1 (X-Porte)	0.857	0.61	0.634	0.622	0.898	0.620	
		Test set 2 (Venue Go)	0.925	0.638	0.549	0.59	0.951	0.606	
		Test set 3 (TE7)	0.927	0.766	0.636	0.695	0.966	0.773	
		Average	**0.903**	**0.67**	0.606	0.635	**0.939**	**0.666**	500.6

Significant values are in bold.

*Aug* augmentation, *SCL* supervised contrastive learning, *Acc* accuracy, *Pre* precision, *Rec* recall; *fps* frame per second.

By comparison, ResNet34 with supervised contrasting learning (SCL) and augmentation showed a mean accuracy of 0.899, a mean precision of 0.606, a mean recall of 0.74, a mean f1-score of 0.666 and a mean AUROC of 0.932, with no significant differences compared with ResNet34 with GRU and augmentation, and a highest inference speed of 910.8 fps (Table 2; Fig. 3b).

### Comparative performances of the classification and segmentation approaches

In the segmentation approach, it was evident that ResNet with deeper layer (50, 101, and 152) was never better than ResNet34, and this is consistent with results obtained in the classification approach. U-Net with ResNet34 as the final model was evaluated the performance based on the highest probability value on the test set by determining an f1-score threshold of 0.88 in the validation set.

The cascaded model with augmentation showed a mean accuracy of 0.903, a mean precision of 0.67, a mean recall of 0.606, a mean f1-score of 0.635, a mean AUROC of 0.939 and a mean 500.6 fps (Table 2; Fig. 3). This segmentation approach showed poorer performance than the classification approach of ResNet34 with augmentation methods.

**Figure 4:**
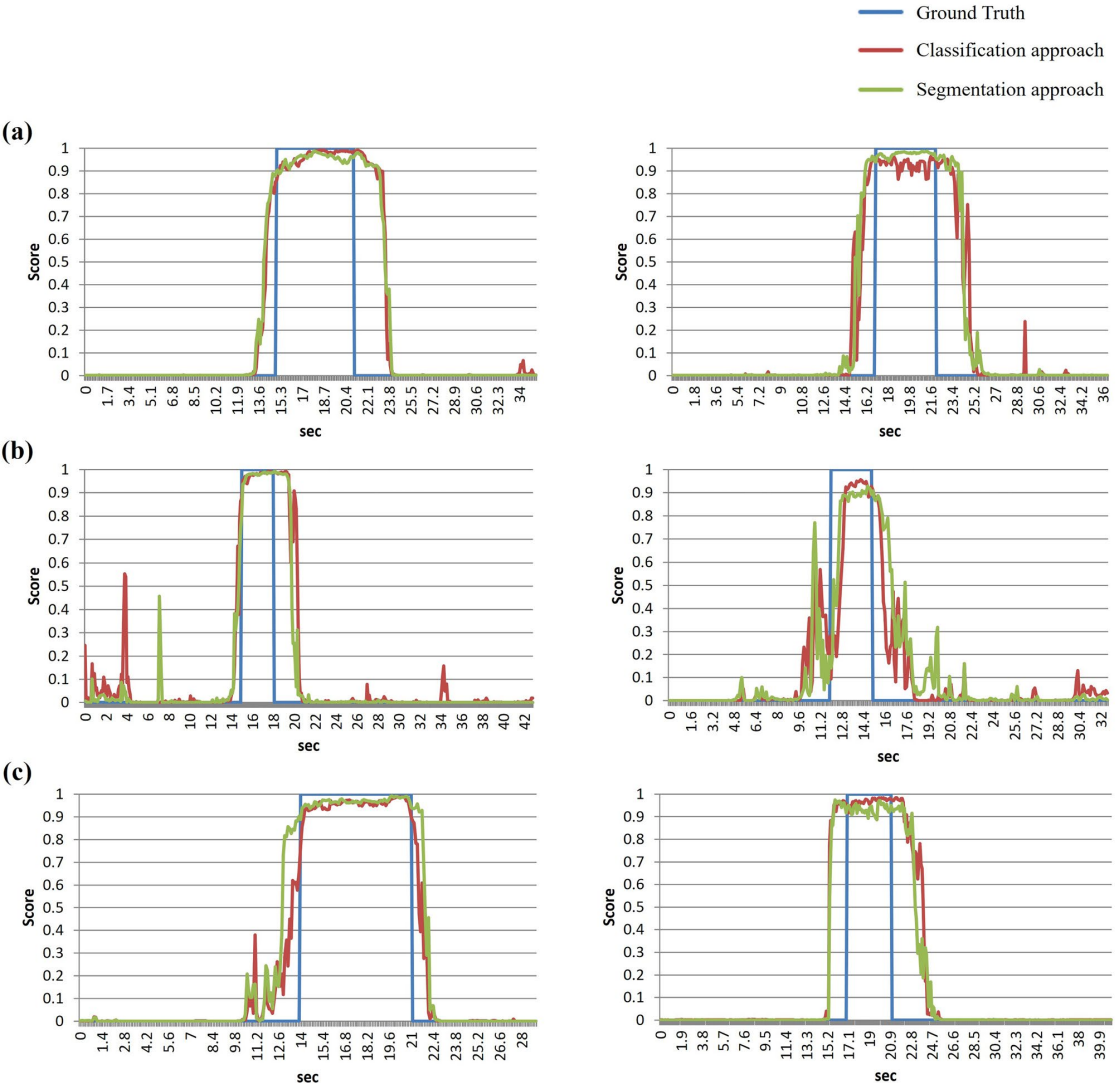
Examples of convolutional neural network prediction and ground truth in each ultrasound video for (**a**) test set 1 (X-Porte), (**b**) test set 2 (Venue Go), and (**c**) test set 3 (TE7).

Figure 2 shows the qualitative results of optimal view determination of ultrasound-guided SCB block using both the classification and segmentation approaches. Application of the trained CNN model during the inference phase enabled visualization of the gradient-weighted class activation mapping (Grad-CAM)^34^ results by overlapping the original ultrasound images. Red colour in the heatmap is indicative of higher chance of finding the optimal view around that area (Fig. 2b). Examples of qualitative results of the CADx system with the ultrasound equipment used are shown in Supplementary Videos 1–6. Figure 4 shows examples of evaluation of the performance of each ultrasound video, consisting of comparisons between the CNN prediction of the optimal view section and the ground truth section. CNN with higher overlapping area between the predicted score and the binary ground truth value is proved to demonstrate a better performance. The threshold values obtained from the validation set for the classification and segmentation approaches were 0.81 and 0.88, respectively. When the threshold was exceeded, the model predicted that test sets 1–3 would provide optimal views.

## Discussion

SCB is a common procedure usually with the CP approach, which has a low risk of causing pneumothorax^8^. Despite this, there have been few studies focused on identifying the optimal view for SCB. To address the current limitations of AI-assisted UGRA for SCB, we proposed a CADx system utilizing deep learning technology. The CADx is likely to be a proper model capable of distinguishing between optimal and non-optimal views for the CP approach with a high AUPRC and a decent inference speed. We also confirmed that the CADx system guarantees considerable robustness across three different ultrasound machines.

The CADx systems have been developed in a variety of fields to assist the optimal view determination such as cardiac^23^, fetal^24^, breast^19^ and thyroid^35^ ultrasound. More specifically, some practical studies about AI-assisted UGRA detect BP at the interscalene level^15,22^ and visualize the relevant anatomic structures in real time^14^. However, to our best knowledge, no studies have described methods for optimal view detection for SCB and our study broadens the applicability of the CADx for SCB.

During labelling in videos, large intra-individual variations even within the optimal views were observed and many optimal images did not show large inter-individual variations when compared with non-optimal views. Nevertheless, the most important ultrasound image characteristics distinguishing optimal from non-optimal views was the finding that BP was located lateral or postero-lateral to the SCA on top of the first rib in optimal views. One concern with the classification approach was the lack of transparency of the process used to determine the outcome, as the CNN model was unable to provide an explanation for the outcome^36^. The Grad-CAM result of the classification approach, however, showed that the heatmap was consistently activated in the area of the first rib. Although it was not intended by clinicians during SCB, the first rib was located in the middle of optimal images. This indirect determination of the method used by the classification model was somewhat consistent with clinical inferences.

The deep learning-based classification and segmentation approaches developed in this study both used ResNet34, enabling the application of a light-weight model for real-time processing in a clinical environment. In general, the number of parameters and the performance of a model tend to be proportional; however, this proportionality was not observed in the ImageNet dataset and other domains such as the chest X-ray dataset^37^. This trend is qualitatively similar to Guo et al.^35^ where even ResNet18 was shown to be highly effective enough to recognize a target image. This indicates that there might be no need for large-sized models in the ultrasound classification task, and also it showed similar results (Supplementary Fig. 4). A simple and cost-effective model without additional information presented here was sufficient for the ultrasound image classification task. This result demonstrates the feasibility of the CADx system in a real-time clinical environment.

In this study, we confirmed that the proposed classification approach shows near real-time performance, and for future application in a real clinic environment, two approaches can be considered. The first is embedding the CAD system into the ultrasound device being used, for which a high-performance GPU system would also need to be installed. Another approach is to apply the CAD system to a screen that captures the output from a split channel of the ultrasound monitor. This has the advantage of allowing for simultaneous comparison of the original ultrasound image and the image to which the CAD system has been applied.

In addition, the CADx system showed similar high performance when evaluating test sets acquired from two other ultrasound imaging devices (Venue Go, TE7) and a test set acquired with the same device as the training set (X-Porte). Thus, the CADx system may be applicable to images acquired with many types of ultrasound machines.

In addition to the cascaded architecture proposed in this study, a model that effectively predicts optimal and non-optimal view segments could potentially be developed using a single architecture through multi-task learning (MTL). However, this approach would require labels for both classification and segmentation, making the labeling process in the preparation of ultrasound video training data burdensome. And also, the optimal view for SCB in this study was defined as where relevant anatomical structures (SA, first rib and BP) are simultaneously observed, they are regarded as quintessential and contain useful information on the optimal view. Therefore, the output of the segmentation network was concatenated with the original ultrasound images to be reused as input to the classification network in cascaded structure.

This study had several limitations. First, since this study is a single centre study and the data were analyzed retrospectively, there may have been selection bias. Multi-center, prospective studies are needed to evaluate the generalizability of the developed model. Second, although the developed CADx systems showed high quality performance, their clinical efficacy has not yet been determined. Although these models have been evaluated using quantitative measures, ​​such as accuracy, f1-score, and fps, it is unclear whether these metrics are associated with clinical efficacy. Thus, real-time clinical application of the CNN model is required to determine whether it improves performance outcomes. Finally, the segmentation approach did not measure the dice coefficient for the test set, as the objective of this study was to distinguish between optimal and non-optimal views for ultrasound-guided SCB, so only the classification performance was evaluated quantitatively.

In conclusion, this study described the development of CADx systems, using both classification and segmentation approaches, which could optimally detect corner pocket images for complete SCB. Both approaches showed high performance in detecting optimal views and functioned well in real-time settings. This proposed method may be applicable to various anatomical structures^14^ and to systems of tracking nerves along their courses and selective trunk identification^38,39^.

### Supplementary Information

Supplementary Figures.

Supplementary Information.

## Data Availability

The data used during the current study are available from the corresponding author on reasonable request.
