# Author Correction: Optimal view detection for ultrasound-guided supraclavicular block using deep learning approaches

**DOI:** 10.1038/s41598-023-46973-5

**Published:** 2023-11-10

**Authors:** Yumin Jo, Dongheon Lee, Donghyeon Baek, Bo Kyung Choi, Nisan Aryal, Jinsik Jung, Yong Sup Shin, Boohwi Hong

**Affiliations:** 1 Department of Anaesthesiology and Pain Medicine, College of Medicine, Chungnam National University and Hospital, 282 Munhwar-ro, Jung-gu, Daejeon, 35015 Republic of Korea; 2 Department of Biomedical Engineering, College of Medicine, Chungnam National University and Hospital, Daejeon, Republic of Korea; 3 Chungnam National University College of Medicine, Daejeon, Republic of Korea; 4 MTEG Co., Ltd, Seoul, Republic of Korea; 5 Biomedical Research Institute, Chungnam National University Hospital, Daejeon, Republic of Korea

Correction to: *Scientific Reports* https://doi.org/10.1038/s41598-023-44170-y, published online 11 October 2023

The Funding section in the original version of this Article was incomplete.

“This work was supported by Chungnam National University Hospital Research Fund, 2020 and was the result of a study on the “HPC Support” Project, supported by the 'Ministry of Science and ICT' and ‘National IT Industry Promotion Agency (NIPA)’.”

now reads:

“This work received support from the Chungnam National University Hospital Research Fund (2020-CF-005), the National Research Foundation of Korea (NRF) grant funded by the Korea government (MSIT) (NRF-2022M3J6A1084843) and was the result of a study on the “HPC Support” Project, supported by the ‘Ministry of Science and ICT’ and ‘National IT Industry Promotion Agency (NIPA)’.”

The original Article has been corrected.

