# Supplementary material for: Optimal view detection for ultrasound-guided supraclavicular block using deep learning approaches
Source: Sci Rep. 2023 Oct 11;13:17209. doi: 10.1038/s41598-023-44170-y (PMC10567700; doi:10.1038/s41598-023-44170-y)
Supplement: Supplementary file 1 — Supplementary Figures. [file 41598_2023_44170_MOESM1_ESM.pdf]

Optimal View Detection for Ultrasound-guided Supraclavicular Block using Deep Learning Approaches

Yumin Jo, M.D., Ph.D.[1*], Dongheon Lee, Ph.D.[2,5*], Donghyeon Baek, B.S.[3], Bo Kyung Choi, Ph.D.[4], Nisan Aryal [4], Jinsik Jung, M.D.[1], Yong Sup Shin, M.D., Ph.D.[1], Boohwi Hong, M.D., Ph.D.[1,5]

[1]Department of Anesthesiology and Pain Medicine, College of Medicine, Chungnam National University and Hospital, Daejeon, Republic of Korea

[2]Department of Biomedical Engineering, College of Medicine, Chungnam National University and Hospital, Daejeon, Republic of Korea

[3]Chungnam National University College of Medicine, Daejeon, Republic of Korea

[4]MTEG Co., Ltd

[5]Biomedical Research Institute, Chungnam National University Hospital, Daejeon, Republic of Korea

*Contributed equally to this work as co-first authors.

Supplementary figure 1.

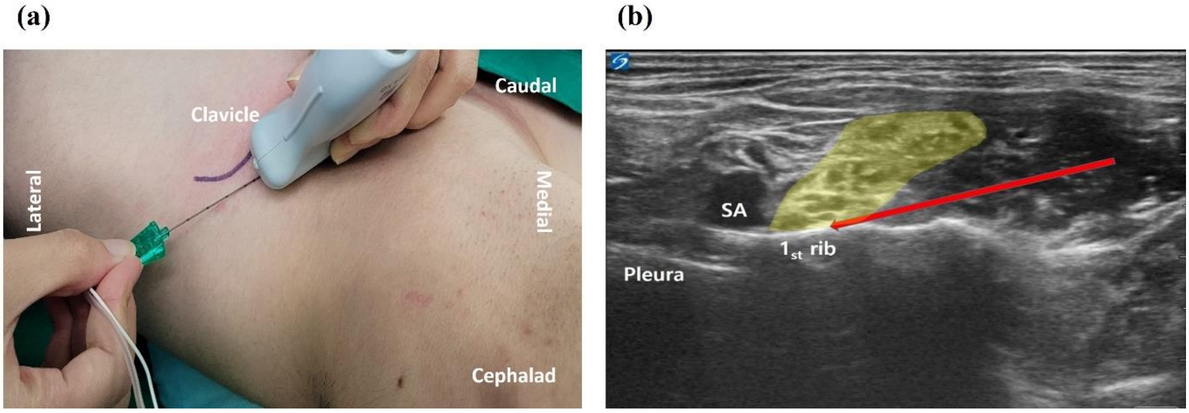

Example of ultrasound-guided supraclavicular brachial plexus nerve block. (a) Placement of the transducer into the supraclavicular fossa just proximal to the clavicle, with caudal tilting to obtain an optimal view. (b) Ultrasound image, showing the brachial plexus (yellow shadow) located postero-lateral to the SA (SA) and on the first rib (red arrow) with the needle position in the corner pocket.

Supplementary figure 2.

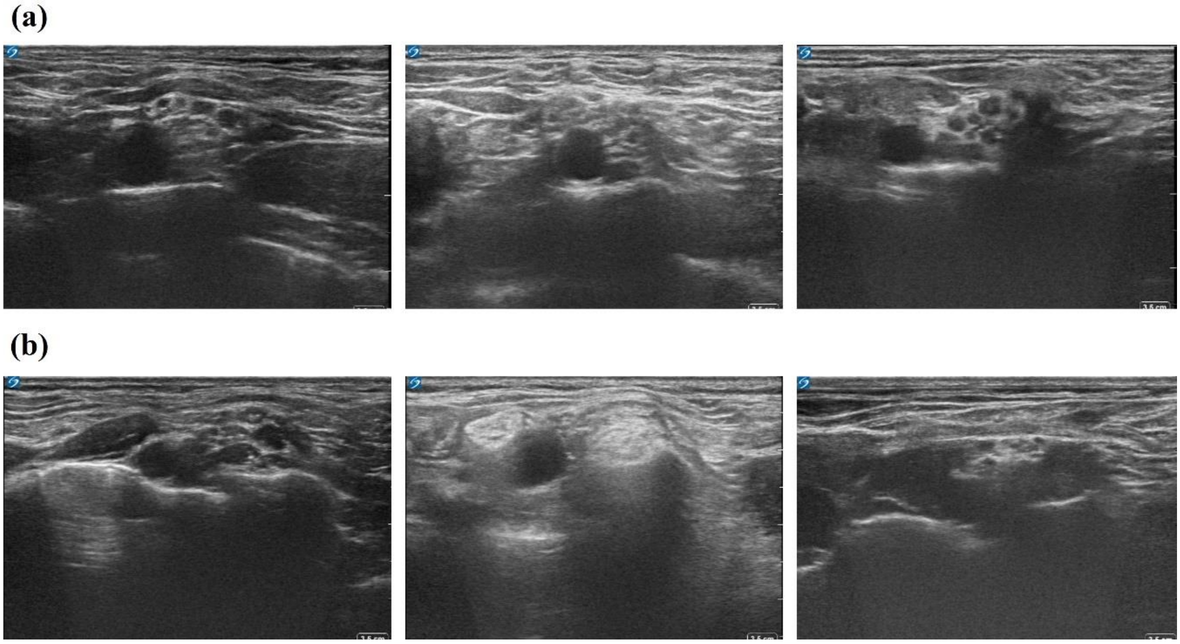

Sample views of an ultrasound-guided supraclavicular block. (a) Optimal views; (b) Nonoptimal views.

Supplementary figure 3.

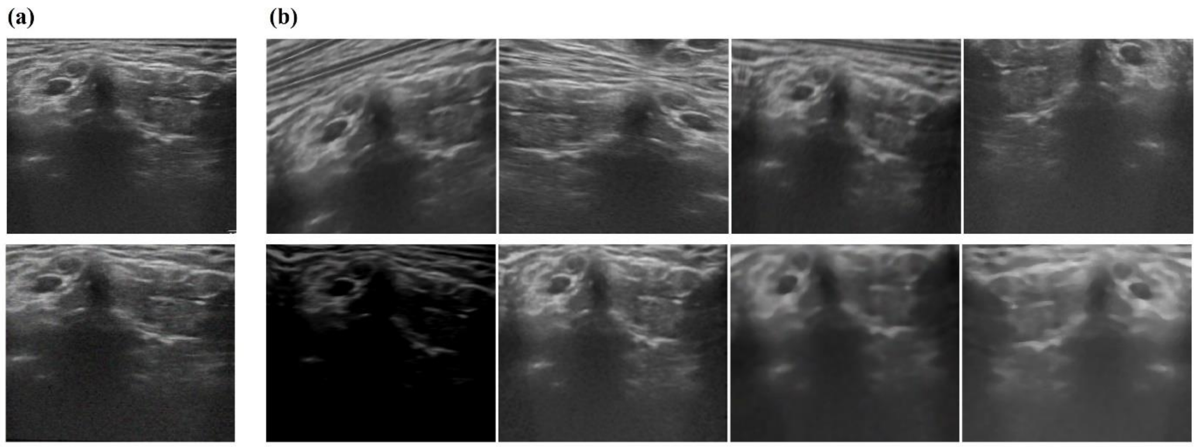

Results of augmentation techniques. (a) Original image. (b) After application of augmentation techniques.

Supplementary figure 4.

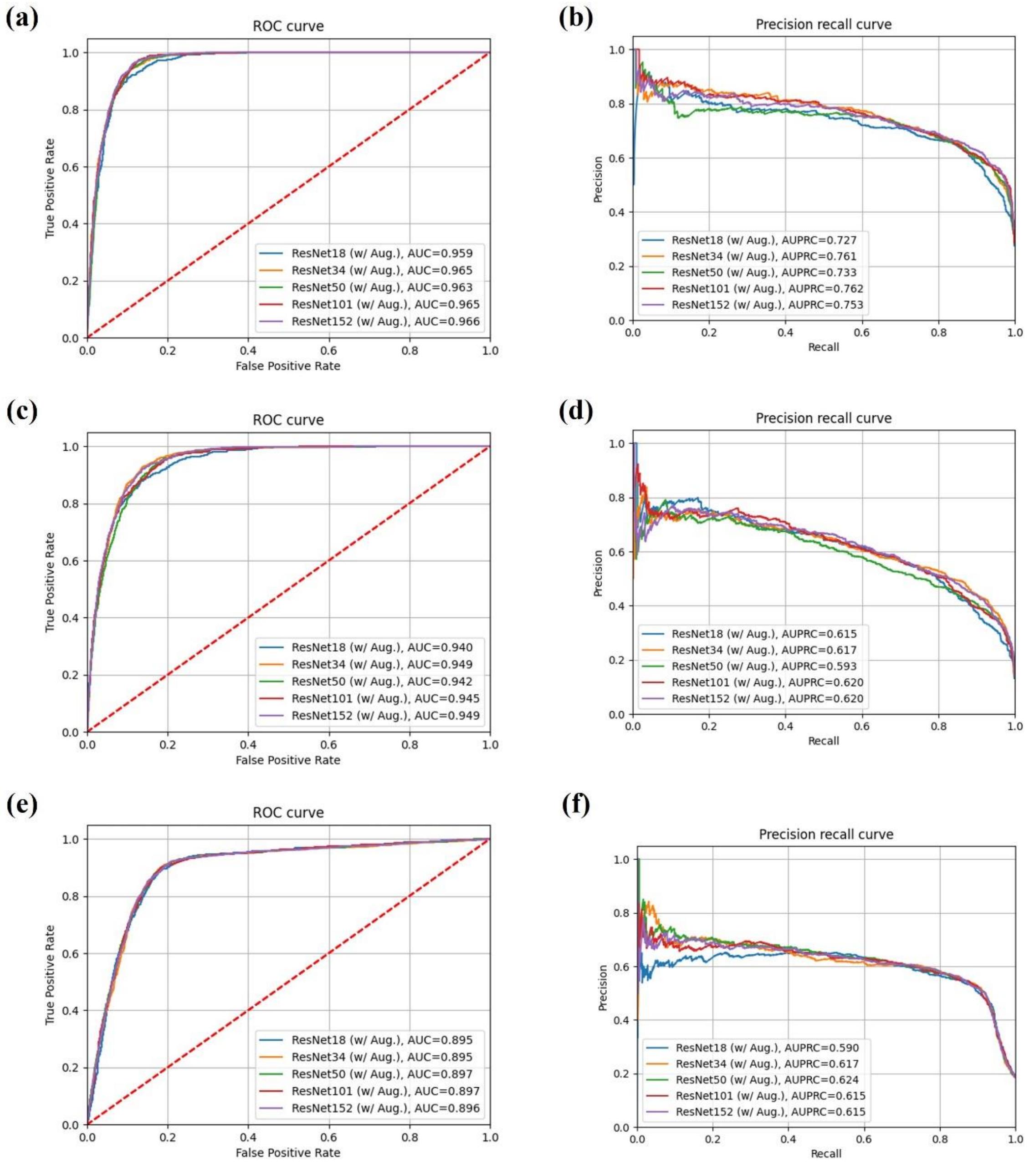

Comparative performances of the classification approaches. (a, c, e) ROC curves of test sets (a) 1 (X-Porte), (c) 2 (Venue Go), and (e) 3 (TE7). (b, d, f) PR curves of test sets (b) 1 (X-Porte), (d) 2 (Venue Go), and (f) 3 (TE7).

Supplementary figure 5.

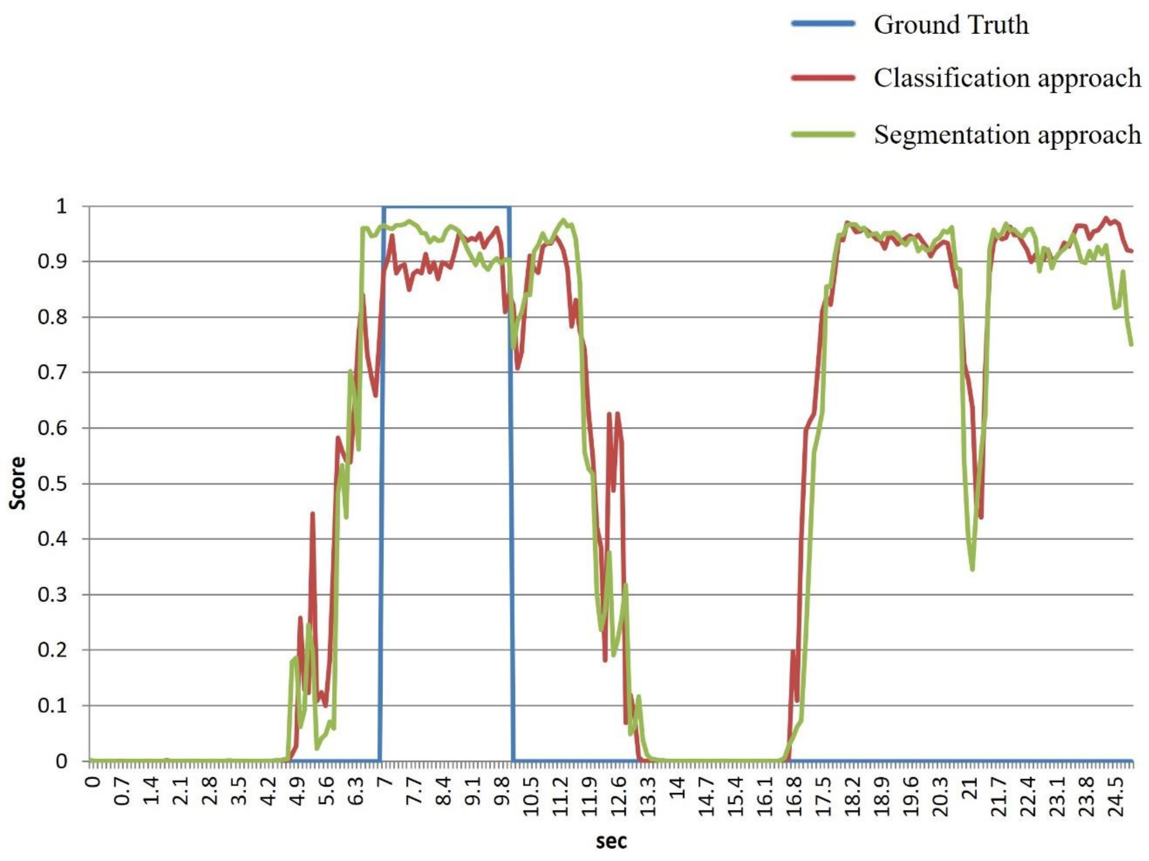

Example of CNN prediction and ground truth in an ultrasound video.

Supplementary figure 6.

Algorithm 1: Method for cropping foreground ultrasound images.

Data: Original ultrasound image I, Kernel K
Result: Extracted ROI image I_{roi}
for *i in height of I* **do**
    for *j in width of I* **do**
        if $I[i,j] > 0$ **then**
            $I[i,j] = 255$;
        else
            $I[i,j] = 0$
        end
    end
end
$I = morphOpen(I, K)$;
$L = findContours(I)$;
$l^* = \arg\max_{l \in \mathcal{L}} contourArea(l)$;
$x, y, w, h = boundingRect(l^*)$;
$I_{roi} = I[y : y + h, x : x + w]$;
return I_{roi};

Example of automated background removal algorithm.
