# Supplementary material for: Optimal view detection for ultrasound-guided supraclavicular block using deep learning approaches
Source: Sci Rep. 2023 Oct 11;13:17209. doi: 10.1038/s41598-023-44170-y (PMC10567700; doi:10.1038/s41598-023-44170-y)
Supplement: Supplementary file 2 — Supplementary Information. [file 41598_2023_44170_MOESM2_ESM.zip › Supplementary videos legends.docx]

**Supplementary videos legends**

Supplementary Video 1. Representative results using the classification approach (X-Porte).

Supplementary Video 2. Representative results using the segmentation approach (X-Porte).

Supplementary Video 3. Representative results using the classification approach (Venue Go).

Supplementary Video 4. Representative results using the segmentation approach (Venue Go).

Supplementary Video 5. Representative results using the classification approach (TE7).

Supplementary Video 6. Representative results using the segmentation approach (TE7).
